# Defining Clinical Malaria: The Specificity and Incidence of Endpoints from Active and Passive Surveillance of Children in Rural Kenya

**DOI:** 10.1371/journal.pone.0015569

**Published:** 2010-12-16

**Authors:** Ally Olotu, Gregory Fegan, Thomas N. Williams, Philip Sasi, Edna Ogada, Evasius Bauni, Juliana Wambua, Kevin Marsh, Steffen Borrmann, Philip Bejon

**Affiliations:** 1 Kenya Medical Research Institute/Wellcome Trust Research Programme, Centre for Geographic Medicine Research, Kilifi, Kenya; 2 Nuffield Department of Medicine, Centre for Clinical Vaccinology and Tropical Medicine, University of Oxford, Oxford, United Kingdom; 3 Department of Clinical Pharmacology, Muhimbili University of Health and Allied Sciences, Dar es Salaam, Tanzania; 4 Department of Infectious Diseases, Heidelberg University School of Medicine, Heidelberg, Germany; Université Pierre et Marie Curie, France

## Abstract

**Background:**

Febrile malaria is the most common clinical manifestation of *P. falciparum* infection, and is often the primary endpoint in clinical trials and epidemiological studies. Subjective and objective fevers are both used to define the endpoint, but have not been carefully compared, and the relative incidence of clinical malaria by active and passive case detection is unknown.

**Methods:**

We analyzed data from cohorts under active and passive surveillance, including 19,462 presentations with fever and 5,551 blood tests for asymptomatic parasitaemia. A logistic regression model was used to calculate Malaria Attributable Fractions (MAFs) for various case definitions. Incidences of febrile malaria by active and passive surveillance were compared in a subset of children matched for age and location.

**Results:**

Active surveillance identified three times the incidence of clinical malaria as passive surveillance in a subset of children matched for age and location. Objective fever (temperature≥37.5°C) gave consistently higher MAFs than case definitions based on subjective fever.

**Conclusion:**

The endpoints from active and passive surveillance have high specificity, but the incidence of endpoints is lower on passive surveillance. Subjective fever had low specificity and should not be used in primary endpoint. Passive surveillance will reduce the power of clinical trials but may cost-effectively deliver acceptable sensitivity in studies of large populations.

## Introduction

Childhood febrile disease is the most common clinical manifestation of *P. falciparum* infection and is the endpoint most commonly used to measure the public health burden of the disease, and to assess the efficacy and effectiveness of preventative interventions such as bed nets [1] or vaccines [2], including early phase trials [3]. Both active and passive surveillance methods have been proposed to assess this endpoint and consensus guidelines on their use in vaccine trials have been published [4]. However, there are no analyses that examine the sensitivity and specificity of febrile malaria case definitions identified by passive case detection, yet this is critical to the accuracy of clinical trials and public health surveillance.

Defining clinical malaria in malaria endemic countries is difficult because individuals may carry parasites without symptoms, and coincidental febrile episodes may have etiologies other than malaria. The malaria-attributable fraction method uses population data to estimate the frequency of true febrile malaria among all febrile cases by fitting the risk of fever as a function of parasite density using a logistic regression model[5]. This method has been widely used under different malaria endemicities, and has become a standard approach for deriving parasite density thresholds to optimize sensitivity and specificity [6], [7], [8], [9], [10], [11].

In active surveillance, individuals are visited regularly (monthly, fortnightly or weekly) and assessed for malaria infection or disease status. Provision is usually made to identify febrile episodes between visits. This additional provision is often referred to as “passive surveillance”, and hence the combination may be called “active and passive surveillance”. However, the likelihood of presentation between visits often rises as a result of the frequent contact [12]. For this reason, and also to avoid confusion, we have described the combination as active surveillance in this paper. The alternative, which we refer to as passive surveillance, relies only on individuals' attendance at health care facilities to identify episodes of malaria.

It has been argued that active surveillance is more likely than passive surveillance to misdiagnose asymptomatic parasitaemia with a coincident, non-malaria cause of fever as a true malaria case, leading to over-estimation of burden of malaria [13]. However, passive surveillance may miss clinical episodes that are treated or resolve spontaneously without presentation to health care facilities[14], [15], [16]. Cohorts under active surveillance often record higher rates of malaria episodes than those under passive surveillance [17], [18] which may be interpreted either as the inclusion of false-positives by the former, or an under-estimation of the true malaria burden by the latter [19], [20], [21]. Estimates of the global burden of malaria disease has relied on cohorts under active case detection [22].

Comparison between surveillance methods can be confounded by the location, malaria endemicity, age of individual being followed and period of follow up. Therefore a formal comparison of the relative incidence of endpoints detected by passive and active surveillance requires matching by these variables, and has not previously been undertaken.

Regardless of the surveillance method used, cases are often defined by malaria parasitaemia in association with either an objective fever (i.e. temperature>37.5) or a subjective fever. Use of subjective fever in the case definition may lack specificity, and has been avoided in some studies[6], but the specificity of this endpoint has not been formally calculated.

We therefore analyzed data across four different cohorts in Kilifi District, Kenya, in order to describe the sensitivity and specificity of different case definitions from active and passive surveillance. We also performed a formal comparison of specificity and incidence of mild malaria endpoints from active and passive surveillance in a sub-group of children who were matched by age, period of follow up and location (implying similar malaria endemicity).

Previous studies have suggested that a threshold of 2,500 parasites per µl adds specificity to the case definition [7], and so we describe and compare estimates with and without this threshold.

## Methods

### Study cohorts and surveillance

**Figure 1:**
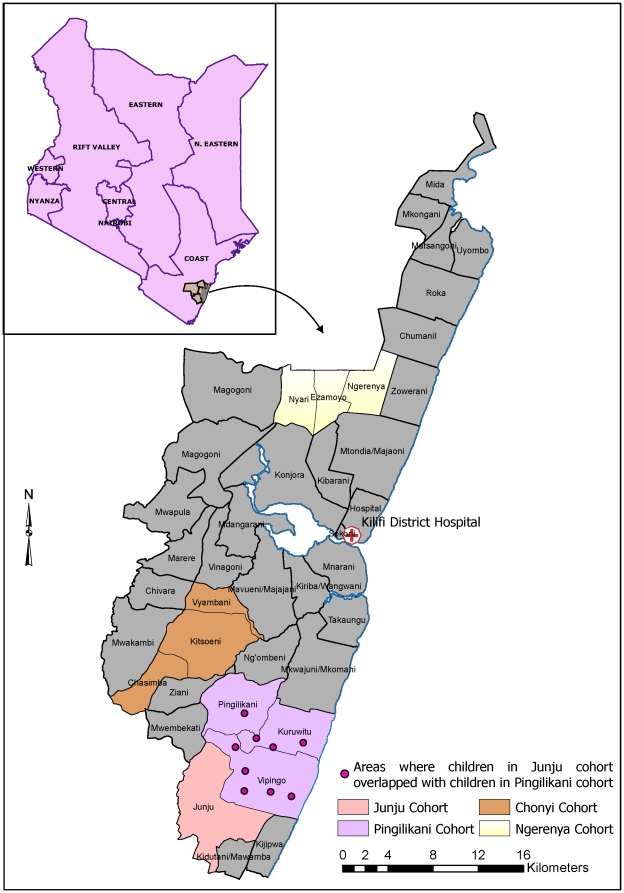
Location of the cohorts used in the study. The map shows the location of the cohorts used in the analysis. Colored regions represent the locations where the cohorts were located. The sub-locations within each location are also shown, some of which have the same name as the cohort.

We analyzed data from four cohorts which underwent different surveillance methods for mild *P. falciparum* malaria, in order to describe the sensitivity and specificity of case definitions within cohorts and compare the specificity and incidences of the endpoints from active and passive surveillance. The cohorts were located in Chonyi, Ngerenya, Junju and Pingilikani sub-locations of Kilifi District, on the coast of Kenya between January 1998 and June 2009 (Figure1). The Junju cohort included children located both in Junju and Pingilikani sub-locations. All cohorts were nested within the wider demographic surveillance system (DSS) which covers an area of about 891 km^2^ around Kilifi District Hospital and involves six monthly re-enumeration visits to about 25,000 households. Junju and Pingilikani have generally been high transmission areas, with moderate transmission in Chonyi, and low transmission in Ngerenya, as evidenced by entomological studies [23] and parasite rates [6], [7]. However, transmission has been falling throughout the period of study [24], [25]. For the purpose of this study we categorized the transmission intensity based on concurrent parasite prevalence in each cohort.

Different follow up and blood slide evaluation protocols were used in each cohort. In the Chonyi and Ngerenya cohorts, clinical malaria episodes were detected using weekly active surveillance implemented over the entire study period [7]. Children with subjective or objective fever (axillary temperature≥37.5°C) had blood samples taken to estimate the parasite density from blood smears. In contrast, in Junju blood smears were done only on children with an objective fever (axillary temp≥37.5°C) and children with subjective fever without elevated temperature were followed 6–12 hours later, and the temperature measurement repeated. Blood smears were made if objective fever was confirmed at this measurement. Clinicians reviewed all children who were unwell but without objective fever. In addition while the parents of the children in Chonyi and Ngerenya were instructed to report to Kilifi District Hospital (20 km away) if the child had any symptoms of disease at any time, in Junju, dispensaries were located within 5 km and trained field workers were available at all times in the villages for passive surveillance. Pingilikani cohort was monitored purely by passive surveillance at the Pingilikani dispensary, where blood smears were done for all children presenting with a complaint of fever (both objective and subjective fever). Sulfadoxine-pyrimethamine was the first line anti-malarial drug used until early 2006 when the artemisinin base combination therapy (artemether-lumefantrine) was introduced throughout Kenya.

### Ethics statement

The details of consent procedures have been published elsewhere [7], [26]. Briefly, in Chonyi and Ngerenya, written informed consent was obtained from parents/guardians of young children and adults from randomly selected homesteads using an approved consent form. In Junju written informed consent was obtained from parents/guardians of children who earlier participated in a non-efficacious malaria vaccine trial. Subsequently the consent was sought for all the newborns from these homesteads. Pingilikani dispensary cohort is part of a wider Demographic Surveillance system with established recruitment process. The clinical records of children in the cohort were obtained by matching their personal identification numbers from anonymised Demographic Surveillance System and dispensary database.

The approval for human participation in these cohorts was given by the Kenya Medical Research Institute Scientific Committee and National Review and the Ethical Committee of the Kenya Medical Research Institute.

### Laboratory investigations

Malaria parasitaemia was determined by examination of blood smears stained with 2% Giemsa solution. For Junju, Pingilikani dispensary, Chonyi and Ngerenya cohort, the number of asexual-stage parasites/200 leukocytes was counted, and parasitaemia was estimated on the basis of an assumed uniform white cell count; 8,000 leukocytes/µL. For members of the Junju cohort located in the Pingilikani sub-location, parasitaemia was estimated on the basis of actual leukocyte count measured for each blood smear. Regression models for the determination of Malaria Attributable Fraction used parasite densities calculated from a single method. Only *Plasmodium falciparum* parasitaemia was included in the analysis.

### Calculation of Malaria Attributable Fraction

Malaria Attributable Fractions were used to estimate the specificity of the malaria endpoints detected by different surveillance methods. Cases were febrile children at the time of surveillance visit. Control cases were afebrile healthy children seen at the cross sectional bleeds during the follow up period. For Pingilikani dispensary cohort, we used controls from Junju cross sectional bleed. Junju is contiguous with and immediately to the south of Pingilikani. Malaria Attributable Fraction was determined by using logistic regression to model the risk of fever as continuous function of parasite density as developed by Smith et al [5]. 
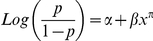



Where p is the probability that a subject with x parasite density has fever and π is the power function of parasite density. The power function maximizes likelihood estimation for the different age groups, cohorts and surveillance methods and was used to model the relationship between fever and parasite density as a continuous function. The power function was re-optimized for each fit of the model, hence the shape of the curve relating parasite density to the probability of fever was allowed to vary for different populations. Confidence intervals were calculated by the bootstrap method using 1000 repeat samplings. Analysis was performed using STATA software (version 9.0; STATA Corp). Only children aged 0–5 years old were included in the analysis of general MAF. Age specific MAF were estimated to investigate variation of MAF with age for each cohort. Children who had objective fever and also were reported to have subjective fever were included in the sub-analysis of “objective fever” but not “subjective fever”.

### Incidence rate

Active surveillance in the Junju cohort located in Pingilikani sub-location overlapped with passive surveillance at Pingilikani dispensary. This overlap applied to the 105 children aged 5–17 months who were under active surveillance between May 2007 and April 2008. In order to define a comparable cohort under passive surveillance at Pingilikani dispensary, we used the demographic surveillance system to identify age- and location-matched children during the same period. However, we did not include children sharing a homestead with a child under active surveillance in this matched cohort, to avoid a possible contaminating effect of the homestead being visited. Incidence rates of malaria clinical episodes were calculated by counting the number of clinical episodes of malaria divided by the total time at risk expressed as total person years at risk.

## Results

The detailed characteristics of each cohort are shown in Table 1. In total there were 7,606 children and 299,189 surveillance visits across all four cohorts. Fever was documented in 19,462 surveillance visits and clinical malaria was diagnosed in 9,219 surveillance visits.

**Table 1:** Baseline characteristics of cohorts used in the analysis.

	Chonyi cohort	Ngerenya cohort	Pingilikani cohort	Junju cohort
Number of children	315	575	6123	488
Follow up period analyzed	1998–2001	1998- June 2006	2003- June 2009	2006- March 2009
Median age in years (IQR) at the start of follow up*	1.8 (3.3)	0.5(2.5)	1.6 (2.1)	2.5 (2.3)
Female %	153 (49%)	292 (51%)	2760 (45%)	202 (41%)
Transmission intensity	High	Low	Moderate	Moderate
Time at risk (years)	480.4	1930.4	25,144.1	864.8
Incidence rate	1.16[1.07–1.26]	0.63[0.6–0.67]	0.16[0.15–0.16]	0.93 [0.87–1.0]
Parasite prevalence#	30.8%	8.8%	18.7%	18.7%
Surveillance methods	Weekly active surveillanceby field worker Passive surveillance at district hospital	Weekly active surveillance by field worker Passive surveillance at district hospital	Passive visits at dispensary	Weekly active surveillanceby field worker Passive surveillance by field workersand at dispensary
Indication for blood smear	Axillary temperature ≥37.5or history of fever in thelast 24 hours.	Axillary temperature ≥37.5or history of fever in thelast 24 hours.	Axillary temperature ≥37.5 or history of fever in thelast 24 hours.	Axillary temperature ≥37.5only
Number of total contact	29,353	178,292	28,384	59,604

# = Parasitaemia prevalence for all cohorts is summed up across the entire follow up period.

* = Only children less than 5 years old are included in the analysis

### MAFs of clinical malaria with objective or subjective fever

Using a subjective history of fever in the case definition was associated with a consistently lower MAF than objectively elevated temperature (temp≥37.5°C) independent of age. This difference was more marked in the higher transmission cohorts (Chonyi and Pingilikani) Table 2. As expected, using a threshold of >2500 parasite/ul increased the MAF for all case definitions. However, among children with subjective fever in Chonyi the MAF was 59% even after applying this threshold (Table 2).

**Table 2:** Malaria attributable fractions of malaria case definitions for any parasitaemia and >2500/uL parasites in the four cohorts.

	Using objective fever(temp≥37.5 C)	Using subjective *but not objective* fever (temp<37.5 C)	Using both case definitions
*Chonyi cohort; active surveillance cohort*			
MAF for density >0/µL (95%CI)	**68% (63%**–**71%)**	**44% (38%**–**50%)**	**56% (52%**–**61%)**
Number of cases	558	1105	1663
MAF for density>2500/µL (95%CI)	**81% (78%**–**84%)**	**59% (53%**–**64%)**	**70% (66%**–**74%)**
Number of cases	456	671	1127
*Ngerenya cohort; active surveillance cohort*			
MAF for density >0/µL (95%CI)	**76% (73%**–**78%)**	**67% (65%**–**69%)**	**74% (72%**–**76%)**
Number of cases	1225	1802	3028
MAF for density>2500/µL (95%CI)	**83% (81%**–**85%)**	**81% (79%**–**83%)**	**83% (81%**–**85%)**
Number of cases	1060	1253	2313
*Pingilikani cohort; passive surveillance only cohort*			
MAF for density >0/µL (95%CI)	**75% (74%**–**76%)**	**40% (39%**–**41%)**	**61% (60%**–**61%)**
Number of cases	3954	3209	7163
MAF for density>2500/µL (95%CI)	**85% (84%**–**86%)**	**54% (53%**–**55%)**	**73% (72%**–**74%)**
Number of cases	2433	1609	4042
*Junju cohort; active surveillance package cohort*			
MAF for density >0/µL (95%CI)	**72% (69%**–**76%)**	NA	NA
Number of cases	809	NA	NA
MAF for density>2500/µL (95%CI)	**85% (83%**–**88%)**	NA	NA
Number of cases	636		

In the Junju cohort, where blood smears were only made on objectively febrile children, on only 8 out of 532 occasions (0.02%) were children with a history of fever but no elevated temperature found to have febrile malaria on a return visit conducted 6–12 hours later. The children who had no objective fever at 6–12 hours follow-up had a similar risk of a subsequent malaria episode as the children (matched by season and age group) who had no history of fever (HR: 0.9 95%CI 0.7–1.5, p value = 0.9).

Older children (2.5–5 years) had lower MAF than younger children (0–2.5 years) which increased after using a parasite threshold of >2500/µL (data not shown). However this difference was less marked in a low transmission cohort (Ngerenya).

### Parasite densities

**Figure 2:**
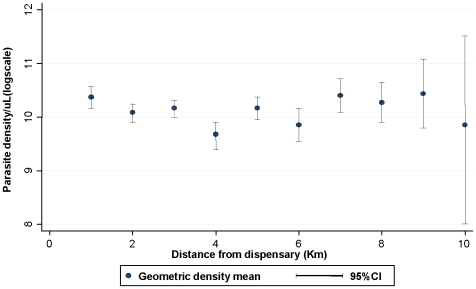
Variation of geometric parasite density mean with distance from Pingilikani dispensary. Variation of geometric mean parasite densities with distance from Pingilikani dispensary. Parasite densities have been converted into log_10_ scale.

Children under passive surveillance in Pingilikani had a significantly higher geometric mean parasite density than children under active surveillance in Junju [10,300/µL:95% CI: 9,800–10,800 versus 4,775/µL:95%CI: 3,900–5,800]. Furthermore, there was no variation in geometric mean parasite density with distance from dispensary for the Pingilikani dispensary cohort within 10 kilometers of the dispensary suggesting that distance to healthcare was not a factor that determined the likelihood of presentation (Figure 2).

### Comparison of active and passive surveillance

Part of the Junju active cohort and the Pingilikani dispensary-based cohort overlapped geographically (Figure 1). Among an age-, time- and location-matched subgroups of these cohorts the Malaria Attributable Fractions were similar (Table 3). However, the incidence of clinical malaria identified by active surveillance was over three times the incidence by passive surveillance. The mean febrile temperature for passive surveillance at dispensary cohort was 38.6°C [95%CI: 38.6–38.7] compared with 38.3°C [95%CI: 38.2–38.4] on active surveillance, although the geometric mean parasitaemia were not statistically significantly different (Table 3).

**Table 3:** Comparison between passive surveillance and active surveillance during follow up from May 2007 to May 2008 in a subset of children aged 5–17months.

	Passive surveillance#	Active surveillance package*
Cohort location	Pingilikani	Junju-Pingilikani
Total number of children	561	105
Total number of contacts	932	3394
Total time at risk (person years)	562.6	91.6
Total malaria episodes ^θ^	58	34
Median contacts (Range)	1.6 (1–9)	32 (2–48)
Geometric mean parasite density (/µL) (95% CI)	14,700(8,700–24,900)	41,000(18,300–92,000)
Children with at least one malaria episode	33	14
Incidence of clinical malaria^θ^	0.10 [95%CI: 0.08–0.13]	0.37 [95% CI: 0.27–0.52]
Malaria Attributable Fraction >0/µL	92% (89%–95%)	88% (81%–96%)
Malaria Attributable Fraction >2500/µL	98% (95%–100%)	94% (87%–100%)

* = The active surveillance package consisted of passive surveillance at dispensary, passive surveillance at community by trained field worker with supervision from study clinician and active weekly surveillance by trained field workers.

θ = Clinical malaria defined as fever (axillary temperature≥37.5°C plus parasitaemia of any density.

# = Passive surveillance package consisted of Passive surveillance at dispensary only. Numbers in brackets are 95% confidence interval unless indicated otherwise.

## Discussion

Clinical malaria is the endpoint most commonly used in the field to measure the efficacy of interventions to prevent malaria, and is often used to assess the public health burden. In a subset of concurrent location- and age-matched cohorts, we found that purely passive surveillance without specific prompts for visits detected about one third of the episodes that would have been identified by active surveillance yet MAFs were similar. Furthermore, cases with subjective fever but no objective evidence (temperature <37.5°C) had persistently lower Malaria Attributable Fractions, even when a parasite density threshold was used.

It is surprising that the difference between rates for active and passive case detection in matched subset of children was not larger. Pingilikani dispensary serves a population of 4000 children under 5, and there had been no contact at the homestead or village level to encourage attendance. In contrast, active surveillance required one local fieldworker per 30–40 children monitored with frequent contact at the homestead level (compared to 1 field worker per >1,000 children in passive surveillance). The large numbers of children using Pingilikani dispensary could be attributed to the availability of adequate staff seven days a week, good malaria diagnostic facilities and a constant antimalarial drug supply in contrast to frequent supply problems reported elsewhere in Kenya [27].

Nevertheless, fewer cases of malaria were identified at the dispensary than would have been seen on active surveillance, and in comparison children brought to the dispensary had significantly higher mean temperatures when they were febrile but similar symptomatic blood stage parasitaemia. This suggests that parents were inclined to postpone presentation at the dispensary until more acute overt illness. This was not simply the parents' inability to identify illness, since when field workers were made highly accessible in the subset of Junju cohort children located in Pingilikani sub-location, the majority of malaria episodes were identified by assessments initiated by the mothers between regular weekly visits. There was no gradient of increasing parasite density with distance from the dispensary, suggesting that the distance parents had to travel with their children did not delay their treatment seeking. We conclude that, in our setting, there is a barrier to approaching medical staff in a health facility that does not exist with more familiar, local field workers. Cases of febrile malaria not presenting to the local dispensary may resolve without treatment or be treated by anti-malarials bought by the parents from shops[28].

Passive surveillance in health care facilities has been reported to be insensitive outside Africa [19], [20] and globally [22]. As malaria incidence falls, and it becomes increasingly important to identify the majority of infections to sustain progress in control [29]. Novel approaches, for instance passive surveillance operated by fieldworkers stationed locally, deserve further consideration and evaluation.

The proportion of fevers attributable to malaria was persistently lower in children with subjective fever than in children with objective fever. Fever diagnosis by parents is unreliable [30], [31]. Almost half of the cases with reported but not objective fever were classified as not “true malaria” (i.e. they represented asymptomatic parasitaemia in febrile patients with other etiologies). Based on our results, the inclusion of reported fever cases in the endpoint of a clinical trial, or in measuring the public health burden of malaria is questionable.

Other factors may be relevant in defining the endpoint of a study. Although the main outcome for most Phase IIb vaccine trials in children would be clinical malaria, cluster randomized trials of transmission blocking vaccines might appropriately include all parasitaemic cases identified regardless of the definition of clinical malaria.

However, approximately half of all cases presenting had only subjective fever, and so the loss in power of the study must be balanced against the need to include endpoints of high specificity. As an illustration, consider a hypothetical example where the incidences of subjective and objective febrile malaria were both 10 per 100 children, and the respective MAFs were 54% and 85% (using a threshold of 2,500 parasites/µL). A study would have 90% power to detect 50% vaccine efficacy on including 1800 children using objective fever alone as the endpoint, falling to 1300 children using objective plus subjective fever. However, the estimate of efficacy would be 43% using objective fever, but 35% using objective fever plus subjective fever.

When we followed-up 532 children with subjective fever in Junju cohort 6–12 hours later, we found only 4% (19/532) developed an objective fever despite not receiving treatment and only 8 (0.02%) had parasitaemia. Furthermore, children who remained afebrile at 6–12 hours of follow-up had a similar risk of subsequent malaria episodes and other adverse events as other children matched by age and time of follow up. This was done in a clinical trial where we carefully monitored children and ensured 24 hour access to health care, and would not be practical for routine healthcare or larger studies, but demonstrates that subjective fever alone does not identify children at a high risk of serious disease.

When estimating MAFs in the Pingilikani cohort, cross-sectional surveys from neighboring Junju were used. Since malaria transmission can vary significantly within short distances [32] using controls from a different area could have biased our MAF estimates. Furthermore controls were from a closely monitored cohort which could underestimate the true population prevalence of asymptomatic parasitaemia. However, these two directly adjacent areas are similar in soil-type, housing and Entomological Inoculation Rate (EIR) [23]. A potential estimation error is likely to non-differentially affect both MAF estimates, and thus, is expected not to bias incidence estimates.

Our results of the comparative analysis between passive surveillance and active surveillance apply in the context of our transmission patterns (seasonal/low to moderate) and age group compared, and extrapolation to other transmission patterns and age groups must be done with caution. Ngerenya and Chonyi cohorts operated identical surveillance methods, but the difference between objective fever and subjective fever was greater in Chonyi (at moderate transmission intensity) than in Ngerenya (at low transmission intensity). We have avoided comparisons between surveillance in Junju and Ngerenya/Chonyi because both the surveillance methodology and transmission intensity are different. Another limitation of our study is its retrospective nature which is prone to unmeasured confounding and bias.

In conclusion, the Malaria Attributable Fractions are similar between active and passive surveillance, and passive surveillance at the dispensary underestimates malaria occurrence substantially. Cases of malaria identified on the basis of reported fever alone should not be included in the primary endpoint of a clinical trial since the specificity of the endpoint is low. Phase III studies intended to lead to marketing licenses should test vaccines in the standard setting of dispensary surveillance, but active surveillance should be preferred in Phase IIb studies. Further studies are needed to examine the potential factors inhibiting attendance at health facilities. Improved malaria surveillance at local levels is particularly important for supporting current malaria control efforts and eventually, its elimination.
