# Correction: Defining Clinical Malaria: The Specificity and Incidence of Endpoints from Active and Passive Surveillance of Children in Rural Kenya

**DOI:** 10.1371/annotation/fd5fdb2c-6cdd-429c-ae5c-24334795fe06

**Published:** 2011-02-15

**Authors:** Ally Olotu, Gregory Fegan, Thomas N. Williams, Philip Sasi, Edna Ogada, Evasius Bauni, Juliana Wambua, Kevin Marsh, Steffen Borrmann, Philip Bejon

The first sentence of the funding section is incorrect. It should read: "Philip Bejon is supported by the NIHR Biomedical Research Centre Oxford."

